# Water Sorption and Desorption of Isolated Cuticles From Three Woody Species With Focus on *Ilex aquifolium*

**DOI:** 10.3389/fpls.2021.728627

**Published:** 2021-09-22

**Authors:** Clara Vega, María Valbuena-Carabaña, Luis Gil, Victoria Fernández

**Affiliations:** Departamento de Sistemas y Recursos Naturales, Universidad Politécnica de Madrid, Madrid, Spain

**Keywords:** cuticle, FTIR, holly, microscopy, leaf surfaces, water loss, cutan

## Abstract

The cuticle is a lipid-rich layer that protects aerial plant organs against multiple stress factors such as dehydration. In this study, cuticle composition and structure in relation to water loss are examined in a broad ecophysiological context, taking into consideration leaf age and side from *Ilex aquifolium* (holly) in comparison with *Eucalyptus globulus* (eucalypt) and *Prunus laurocerasus* (cherry laurel). Enzymatically isolated cuticular membranes from holly leaves were studied under three treatment conditions: natural (no chemical treatment), after dewaxing, and after methanolysis, and the rate of water loss was assessed. Structural and chemical changes were evaluated using different microscopy techniques and by Fourier transform infrared (FTIR) spectroscopy. The potential mechanisms of solute absorption by holly leaves were additionally evaluated, also testing if its prickly leaf margin may facilitate uptake. The results indicate that the treatment conditions led to structural changes, and that chemical composition was hardly affected because of the occurrence of cutan. Structural changes led to more hydrophilic adaxial surfaces, which retained more water and were more efficient than natural cuticles, while changes were not significant for abaxial surfaces. Across natural cuticles, age was a significant factor for eucalypt but not for holly. Young eucalypt cuticles were the group that absorbed more water and had the lowest water loss rate. When comparing older leaf cuticles of the three species, cherry laurel was found to absorb more water, which was, however, lost more slowly, compared with the other species. Evidence was gained that holly leaves can absorb foliar-applied solutes (traced after calcium chloride application) through the adaxial and abaxial surfaces, the adaxial mid veins, and to a lower extent, the spines. In conclusion, for the species examined, the results show variations in leaf cuticle composition and structure in relation to leaf ontogeny, and water sorption and desorption capacity.

## Introduction

The cuticle is a protective epidermal layer of plants, located at the interface between plant organs and the surrounding environment (Kerstiens, 1996). The chemical and structural nature of the cuticle have been explained though different models over the years (Von Mohl, 1847; Holloway, 1982; Jeffree, 2006), but recent approaches have suggested to consider the cuticle as a lipidized part of the epidermal cell wall (Domínguez et al., 2011; Guzmán et al., 2014a; Philippe et al., 2020). The cuticle is mainly composed of a cutin/cutan lipid polymer matrix, cell wall polysaccharides, and associated soluble lipids, also known as cuticular waxes (Holloway, 1982; Jeffree, 2006).

The two major components of the lipid polymer matrix are cutin [e.g., main compound in *Lycopersicon esculentum* (*Solanum lycopersicum*) fruit cuticles; Segado et al., 2016] and cutan (the most abundant lipid polymer in, e.g., *Beta vulgaris*; Nip et al., 1986a), or a mixture of both (e.g., *Prunus laurocerasus*; Tegelaar et al., 1991). Cutin is a biopolyester formed by polyhydroxy fatty acids, while cutan is thought to be an ether-linked network of methylene chains, double bonds, and carboxyl groups (McKinney et al., 1996; Villena et al., 1999; Jeffree, 2006). Recent studies suggest that cutan monomers are composed of very long aliphatic chains with different functional groups and a polymeric core of aromatic moieties (Leide et al., 2020). However, because of the high molecular weight and complexity of cutan, its chemical composition is still not fully characterized (Fich et al., 2016; Díaz et al., 2020). Cutan has also been described according to its resistance to degradation by increasingly harsh chemical treatments (Heredia-Guerrero et al., 2014; Leide et al., 2020).

Cutan presence was first reported in detail in *Agave americana* and *Clivia miniata* (Schmidt and Schönherr, 1982; Nip et al., 1986b; Villena et al., 1999), and since then, other species have been tested (Tegelaar et al., 1991; Mösle et al., 1998; Guzmán-Delgado et al., 2016). Cutan has been related to drought adaptation, as it enhances the hydrophobic nature of cuticles (Boom et al., 2005) and might be a target candidate to account for the rigid appearance of some cuticles (Bargel et al., 2006). Furthermore, cutan-containing species are thought to follow strategies such as CAM photosynthesis or a thicker cuticle, which would prevent water loss more effectively. However, thick evergreen leaves or succulence alone do not correlate with cutan presence (Boom et al., 2005; Gupta et al., 2006).

Plant cuticles are also composed of polysaccharides and additional non-polymer lipids, such as waxes (soluble cuticular lipids) (Jeffree, 2006). The inner side of the cuticle is rich in cellulose, hemicellulose, and pectin, which are the most abundant epidermal cell wall polysaccharides (Guzmán et al., 2014a,b; Segado et al., 2016; Philippe et al., 2020). Soluble lipids are located either on the cuticle surface (epicuticular waxes) or distributed within the cuticle (intracuticular waxes) (Jeffree, 2006). A mixture of different long-chain aliphatic molecules, such as alkanes, alcohols, aldehydes, fatty acids, and esters, are present in the cuticle, together with variable amounts of cyclic compounds (Jetter and Riederer, 2016). There is controversy about the role of intra- vs. epi-cuticular waxes as barrier to leaf transpiration (Jetter and Riederer, 2016; Zeisler-Diehl et al., 2018; Zhang et al., 2021), but recent evidence suggests that their importance may be specific to leaf side (Zhang et al., 2020) and species, which respond plastically to drought (Chen et al., 2020; Sanjari et al., 2021). Additionally, variable amounts of phenolic compounds have been recovered in the cuticle of different organs and plant species (Karabourniotis and Liakopoulos, 2005; España et al., 2014).

The genus *Ilex* is present throughout temperate and tropical regions worldwide (Galle, 1997). The only species present in Spain is *Ilex aquifolium* L. (English holly or holly), an evergreen broad-leaved, small dioecious tree. Holly inhabits northern areas as well as some locations in central mountain ranges where the conditions are more humid. This species has been described to have an all-reticulate thick leaf cuticle (Holloway and Baker, 1970), with fibrils reaching the outer surface without a distinct cuticle proper (CP) (Holloway, 1982; Jeffree, 2006). Cuticle morphological characteristics (Jeffree, 2006), cutin description (Holloway and Baker, 1970), cuticle water loss (Burghardt and Riederer, 2003), and cuticle mechanical properties (Khanal et al., 2013) have been examined in previous reports. However, various aspects related to holly leaf cuticle composition, structure, and permeability are not fully understood to date.

The cuticle can be considered a composite membrane made of hydrophobic lipids (chiefly cutin/cutan, waxes, and minor phenol amounts) and hydrophilic polysaccharides (Fernández et al., 2016). The bi-directional transport of water and solutes across the cuticle has been associated with the occurrence of polysaccharides (Chamel et al., 1991; Reina et al., 2001; Riederer, 2006). However, the nano-structural arrangement remains unclear (e.g., Philippe et al., 2020; Segado et al., 2020). Furthermore, environmental factors such as temperature and/or relative humidity (RH) will affect the physicochemical properties of cuticle components (Schönherr and Schmidt, 1979; Domínguez et al., 2011), affecting water transport (i.e., transpiration and water absorption) across the epidermal cell wall (Fernández et al., 2021).

Water deposited onto the leaves after rain, dew, or fog exposure will interact with the surface and condense as films or drops depending on wettability (Fernández et al., 2021). Leaf water condensation has great importance in arid and semiarid regions and can contribute to fog harvesting and water delivery to the roots (Fernández et al., 2021), helping plants cope with water shortage (Malek et al., 1999; Holanda et al., 2019). The site of study, Montejo forest, is located in a valley, which likely causes dew formation during nocturnal cooling (Merinero et al., 2015). Some studies have demonstrated that spines can direct dew from their tips to their bases to help moisturize the plant (Malik et al., 2016), so we aim to evaluate if holly spines may play a role in retaining dew moisture, thus helping the plant to cope with dry season environmental conditions.

The main objective of this study was to examine the potential link between cuticle composition and structure in relation to water loss in a broad ecophysiological context, taking into consideration leaf age and side from holly in comparison with other two species: *Eucalyptus globules* Labill. (blue gum eucalypt or eucalypt) and *P. laurocerasus* L. (cherry laurel). Both sides of young and old cuticles subjected to treatments were evaluated to estimate their water loss rate. Finally, the potential mechanisms of solute absorption by holly leaves were evaluated, also testing if its prickly leaf margin may facilitate foliar absorption.

## Materials and Methods

### Plant Material and Experimental Conditions

Holly leaves were collected in “El Hayedo de Montejo” forest, approximately 90 km north of Madrid, in central Spain (3°30′W, 41°07′N). Montejo is a sub-Mediterranean beech-oak mixed forest, protected according to regional laws. As an average (1994–2019 record), mean annual temperatures correspond to 10°C and annual precipitations to 876 mm. Maximal rainfall occurs in spring (May) and autumn (November) and there is a dry summer season in June, July, and August, where the average precipitation and temperature values are 153.7 mm and 16.7°C (see González, 2015, for 1994–2013 period; 2013–2019 period is unpublished data).

Blue gum eucalypt and cherry laurel leaves were collected from the Forest Engineering School Arboretum (Technical University of Madrid, Spain). Young leaves (sprouted in the year of collection) and old leaves (sprouted in previous years) were collected and taken to the laboratory. Then, leaf margins and mid ribs were removed with a scalpel. For cuticle isolation, leaf sections were immersed in a solution of 5% cellulase, 5% pectinase (Novozymes, Bagsvaerd, Denmark), 1% polyvinylpyrrolidone (Sigma–Aldrich, Munich, Germany), and 2 mM sodium azide set to pH 5 with sodium citrate (Guzmán et al., 2014b) for 1 month. The adaxial and abaxial sides of the leaves were studied separately. Cuticular waxes were removed from the isolated cuticles (500 mg) using a 2:1-(v/v) chloroform: methanol mixture for 24 h at 23°C and 45 rpm speed in an orbital shaker. We used a fraction of the dewaxed cuticles (150 mg) for the methanolysis procedure, as described by Graça and Pereira (2000) for a combined dewaxing-methanolysis treatment.

### Electron Microscopy

Adaxial and abaxial cuticles isolated from the old and young leaves were examined either naturally (i.e., directly after enzymatic isolation) or after chemical treatment (named as D: dewaxed; M: dewaxed and methanolysis). The spines occurring in the leaf margin were analyzed by different microscopy techniques. Scanning electron microscopy (SEM) was performed to explore surface features, while transmission electron microscopy (TEM) enabled us to observe cell wall ultrastructure in epidermal cross sections. For simplicity, cell walls were analyzed in terms of lipidic layer and polysaccharides layer (Fernández et al., 2016). Light optical microscopy (LM) was performed (Zeiss Axioplan II; Carl Zeiss, Jena, Germany) to examine the epidermal cell wall and leaf mesophyll cross sections, and to measure the thickness of different cell wall layers. Cuticle thickness was measured with a micrometer (4,000/F; Baxlo, Barcelona, Spain) (two repetitions per sample).

For SEM and TEM observations, the cuticles were cut into 4 mm^2^ pieces and fixed in 2.5% glutaraldehyde-4% paraformaldehyde (both from Electron Microscopy Sciences [EMS], Inc. Hatfield, PA, United States) for 6 h at 4°C, rinsed in an ice-cold phosphate buffer, pH 7.2, four times within a period of 6 h, and left overnight. The samples for SEM analysis were then dehydrated in an increasing absolute ethanol (Sigma–Aldrich, Munich, Germany) series (30, 50, 70, 80, 90, and 100%; three times for each concentration). They were subsequently subjected to critical point drying (Leica EM CPD300; Leica Microsystems, Wetzlar, Germany). Prior to observation, the samples were gold-sputtered and then examined with a JEOL JSM-6400 microscope (JEOL Ltd., Tokyo, Japan).

For TEM, fixed tissues were also rinsed in the ice-cold phosphate buffer, pH 7.2, four times within a period of 6 h and left overnight. Next morning, the samples were post-fixed for 1.5 h in a 1:1 water and 2% osmium tetroxide (TAAB Laboratories, Berkshire, United Kingdom) solution containing 3% potassium ferrocyanide (Sigma-Aldrich, Munich, Germany). Then, they were washed with distilled water (three times), dehydrated in a graded series of acetone 30, 50, 70, 80, 90, 95, and 100% (twice, 15 min for each concentration) and embedded in acetone-Spurr's resin (TAAB Laboratories, Berkshire, United Kingdom) mixtures (3:1, 2 h; 1:1, 2 h; 1:3, 3 h) and in pure resin overnight (kept at 25°C). Pure resin sample embedding was carried out in blocks that were incubated at 70°C for 3 days. Finally, semi-thin sections were cut, mounted on nickel grids, and post-stained with Reynolds lead citrate (EMS, Inc., Hatfield, PA, United States) for 5 min prior to observation with a JEOL JEM 1010 electron microscope (JEOL Ltd., Tokyo, Japan) at 80 kV and equipped with a CCD camera (Megaview II, Olympus, Japan) (Bahamonde et al., 2018).

Image analysis was carried out with the ImageJ1.52a software (National Institutes of Health, Bethesda, MD, United States) to measure stomatal density and stomatal percentage in total abaxial surface and cuticle and epidermal cell wall thickness of all the groups. Measurements were carried out at periclinal areas on 10 micrographs per sample, with 20 repetitions.

### Fourier Transform Infrared Spectroscopy

Fourier transform infrared spectroscopy is a technique that has proven to be useful for cutane valuation in plant cuticles (Johnson et al., 2007; Fernández et al., 2011; Leide et al., 2020); thus, we used it to study holly cuticle composition. Infrared spectra of all the groups were obtained with an attenuated total reflectance (ATR) accessory (MIRacle ATR; PIKE Technologies, Madison, WI, United States) coupled to an FTIR spectrometer (Nicolet Nexus 670/870; Thermo Fisher Scientific, Waltham, MA, United States). The spectra of the samples were recorded in transmission mode in the range 4,000–400 cm^−1^ (with 4 cm^−1^ resolution accumulating 64 scans) and were analyzed with the Omnicv4.1b (Thermo Fisher Scientific, Waltham, MA, United States) software (Guzmán-Delgado et al., 2016). Both sides of the young and old cuticles by the different treatments (isolated intact cuticles, dewaxed cuticles, and post-methanolysis cuticles) were analyzed.

The esterification index is an indicator of how esterified a cutin matrix is, calculated as the ratio between the ester functional group ν(C=O) and the asymmetric vibration of the methylene functional group reflecting the cross-linking of cutin monomers ν_*a*_(CH_2_). The index is directly related to the cross-linking of cutin, and high values of this ratio imply a higher esterification degree (Benítez et al., 2004; Girard et al., 2012; Heredia-Guerrero et al., 2014).

### Water Loss

The cuticles were weighed after dehydration and after immersion in distilled water for 1 h. Subsequently, the surface of the rehydrated cuticles was gently blotted with soft paper tissue to remove superficial water, and the cuticles were left to air dry, controlling RH and temperature throughout the measuring process. Re-hydrated cuticular water loss was gravimetrically monitored after surface water removal, every 2 min for the first 30 min, every 5 min for the next hour, and every 10 min during the last hour. Measurements were taken for the 12 different groups: young and old leaf cuticles, both adaxial and abaxial side cuticles, and cuticles subjected to different treatments: untreated, dewaxed, and combined dewaxed-methanolysis (two repetitions per group).

For analysis, we adjusted weight-time data to an exponential decay model. The variables studied were: coefficient beta (β), which represents the slope of the decay model, being lower when the water loss curve drops more quickly; Tseca, which is the time (in min) required to reach the asymptote in the decay model; and absorbed, which is the water absorbed in an hour of being submerged in relation to dry weight (%). We also evaluated if temperature or RH influenced the data.

First, we evaluated how the treatments affected water loss in the holly cuticles. Then, the rate of water loss in the natural holly cuticles was compared with that in the eucalypt and cherry laurel cuticles. Blue gum eucalypt lacks cutan (Guzmán et al., 2014a), while cherry laurel contains both cutin and cutan. Cutan is associated with the residue obtained after cuticle solvent extraction and acid-catalyzed transesterification (Tegelaar et al., 1991; Leide et al., 2020). The adaxial eucalypt cuticles were compared with the holly cuticles to study which aspects may vary in the absence of cutan, taking age into account. Furthermore, holly, cherry laurel, and eucalypt adaxial old cuticles were compared to assess species factor.

### Foliar Solute Absorption

We aimed to determine if different holly leaf areas, such as spines, may contribute to the process of solute absorption. First, we used a contact angle meter (DSA 100; Krüss, Hamburg, Germany) to observe the wettability by the water of drops deposited onto different leaf areas, such as spines. Second, using Ca-chloride (Sigma-Aldrich, Munich, Germany) as tracer, we evaluated spine solute absorption in comparison with other leaf parts, namely, the adaxial lamina, abaxial lamina, and adaxial mid vein, by implementing the procedure described by Bahamonde et al. (2018). In brief, leaves were selected and marked in two adult individuals, also selecting a group of untreated control leaves. Early in the morning (around 8 a.m.), using a micro-syringe, drops of approximately 3–4 μl 150 mM CaCl_2_ were applied on leaves still attached to the trees, carefully depositing them onto the mid nerves and spines, but using a brush for both lamina surfaces. After 24 h, the Ca-treated and untreated leaves were detached from the trees and were taken to the laboratory. They were thoroughly washed in an acidulated (0.1 N HCl) 0.1% detergent solution, followed by abundant tap water and two baths of distilled water. The washed leaves were then left to dry at ambient temperature for 1 h, and were subsequently dried in a stove at 50°C for 72 h. The dry leaves were ground to powder and analyzed by inductively coupled plasma-optical emission spectrometry (ICP-OES) to determine tissue Ca concentrations in the treated and untreated holly leaves.

### Data Analysis

Statistical analyses were carried out using the R software (R Core Team, 2013). The data analysis included normality and homoscedasticity tests for the different traits evaluated. Normality was assessed by Shapiro–Wilk test (Razali and Wah, 2011) and homoscedasticity by Fligner–Kileen test. If data were normal and homoscedastic, an ANOVA and a *post-hoc* Tukey's HSD test would be conducted. Otherwise, a non-parametric Kruskal-Wallis test and a pairwise Willcoxon test would be carried out to evaluate differences among the groups.

## Results

### Leaf Structure and Surface Topography

**Figure 1:**
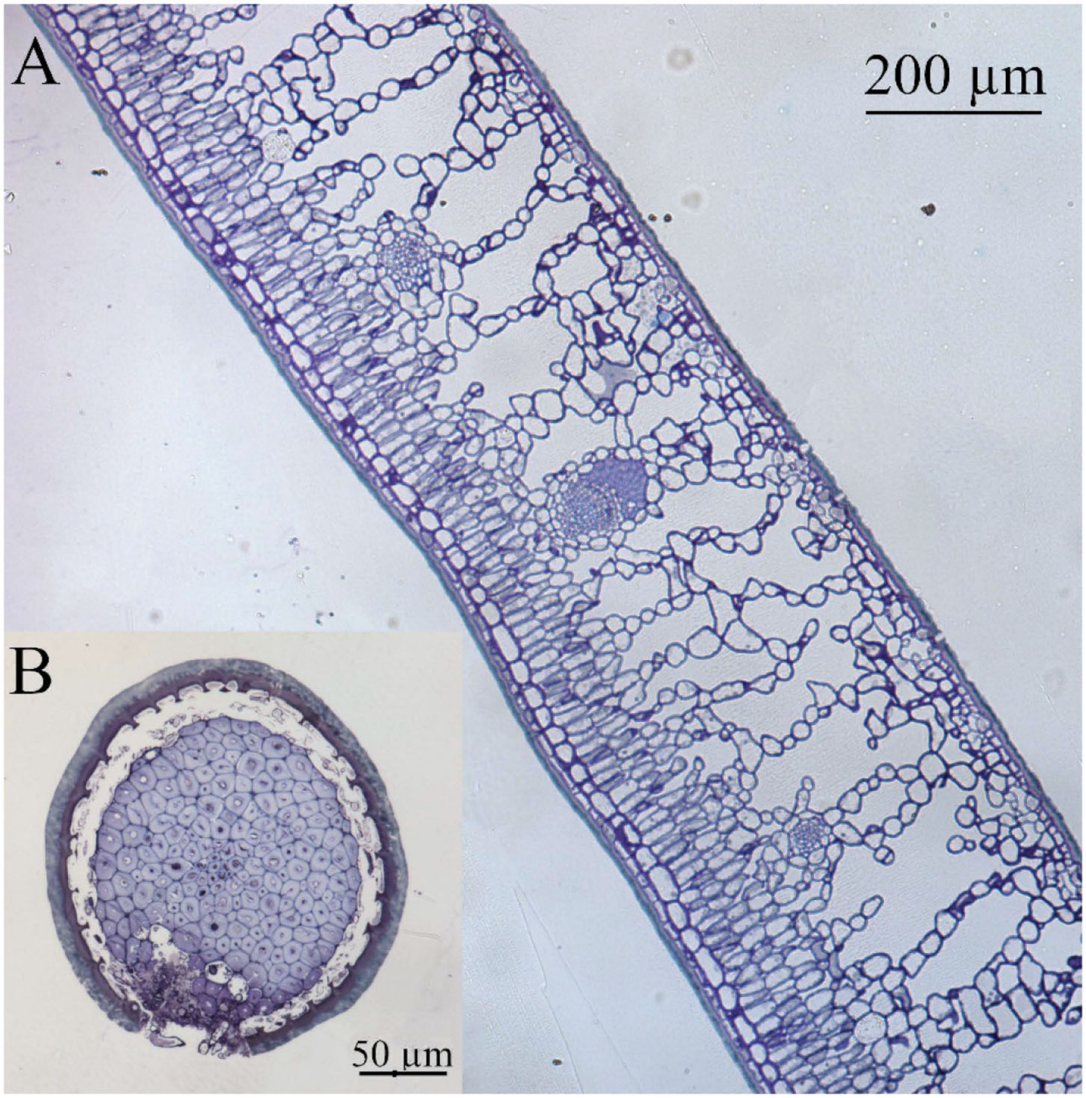
Light microscopy cross section of **(A)** holly leaf and **(B)** spine, both showing a thick cuticle as interface with the surrounding environment. Tissues were stained with Toluidine blue.

**Figure 2:**
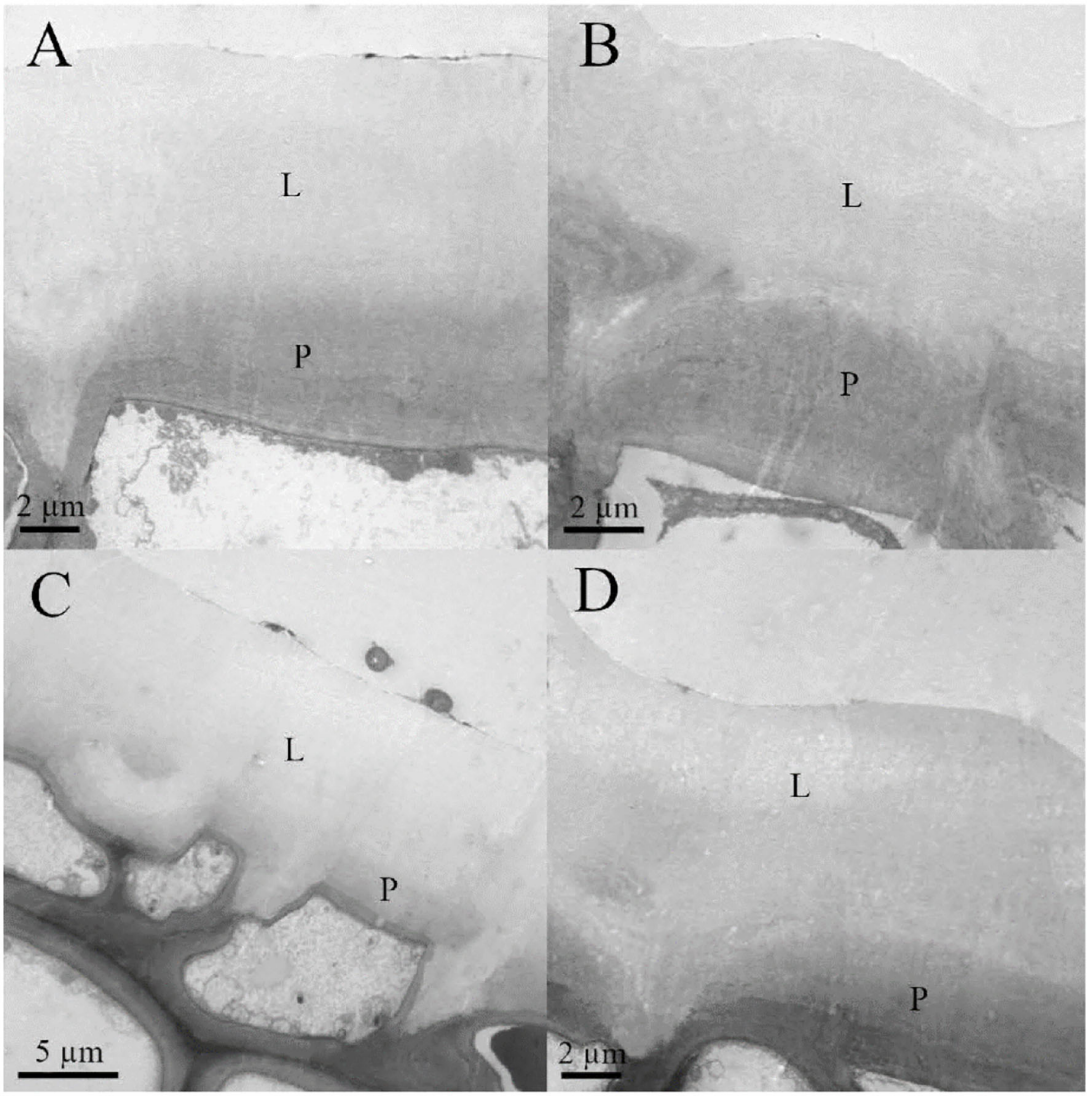
Transmission electron microscopy (TEM) micrographs of **(A)** adaxial young, **(B)** abaxial young, **(C)** adaxial old, and **(D)** abaxial old English holly leaves. Lipidic fraction (L) and polysaccharides fraction (P) of the cell wall are shown.

Leaf thickness had an average value of 442.29 ± 11.33 μm, spongy mesophyll was arranged in columns with large intercellular spaces, and there was a two-to-three-layer palisade parenchyma. A hypodermis is also present in the adaxial side under the epidermis (Figure 1). Regarding cell wall structure, the old leaves had a thicker lipidic layer, while the younger leaves had a thicker layer of polysaccharides (Figure 2). Furthermore, cell wall width values of the old leaves are more dispersed, with the adaxial side being higher than the abaxial side (Table 1). Stomatal leaf density did not significantly vary with age, having young leaves an average of 150.39 ± 20.77 while old leaves had 151.22 ± 15.74 stomata mm^−2^.

**Table 1:** Thickness of different external epidermal cell wall areas.

**Side**	**Age**	**Lipidic fraction (μm)**	**Polysaccharide fraction (μm)**	**Total cell wall (μm)**
Adaxial	Young	7.66 ± 0.39b	4.51 ± 0.40a	12.64 ± 0.37a
	Old	11.80 ± 1.41a	0.70 ± 0.20b	12.45 ± 1.47ac
Abaxial	Young	6.14 ± 0.78b	4.45 ± 0.60a	10.60 ± 0.50b
	Old	8.01 ± 0.84a	3.02 ± 1.36a	11.04 ± 1.21bc
Differences	Age	^***^	^***^	
	Side	^**^		^***^

*Data are means ± SD (n = 30). Within columns and for the same species, values marked with different letters are significantly different according to Tukey's HSD test (P ≤ 0.05)*.

Significance codes: 0 “^***^” 0.001 “^**^” 0.01 “^*^” 0.05 “·” 0.1 “ ” 1.

The prickly part of the leaves (i.e., the spines) has an epidermis covered with a cuticle of similar appearance to the one of the leaf lamina (Figure 1). Spine epidermal cells are separated from a ring of stone cells (sclerenchyma, which surrounds the vascular bundle), by an area of intercellular space. Hence, the main fundamental tissue present in the spines is sclerenchyma in the form of stone cells surrounding the vascular bundle.

### Cuticle Response to Treatments

The treatments significantly affected cuticle structure and composition. The thickness of the natural cuticles (0.032 ± 0.007 mm) decreased by half after dewaxing (0.018 ± 0.008 mm) and by approximately another half after methanolysis (0.008 ± 0.004 mm). Age also affected natural cuticle thickness, increasing adaxial side thickness (from 0.030 to 0.037 mm) but leading to a decrease in the thickness of the abaxial side (from 0.036 to 0.026 mm), which was more affected by dewaxing.

Furthermore, SEM images showed that adaxial cuticle dewaxing (i.e., wax removal, see the gray areas between white colored cell walls, Supplementary Figure 1) was more effective in the young leaves than in the older ones. Methanolysis caused a similar effect on cuticles isolated from both the old and young leaves, which acquired a hollow structure associated with an epidermal cell contour (Supplementary Figure 1). The abaxial cuticle structure was also affected by chemical treatment, mainly by the removal of cuticle materials in patches and especially in areas around stomata. However, after dewaxing and methanolysis, waxes and ester links were not completely removed, as derived from the different colors observed in residual patches (Supplementary Figure 2).

Regarding cuticle composition, a higher proportion of material was extracted by dewaxing (from 24 to 29%) compared with the effect of methanolysis (from 3 to 8%), this proportion being higher for the abaxial leaf cuticles compared with the adaxial ones.

### Spectroscopic Characterization

**Figure 3:**
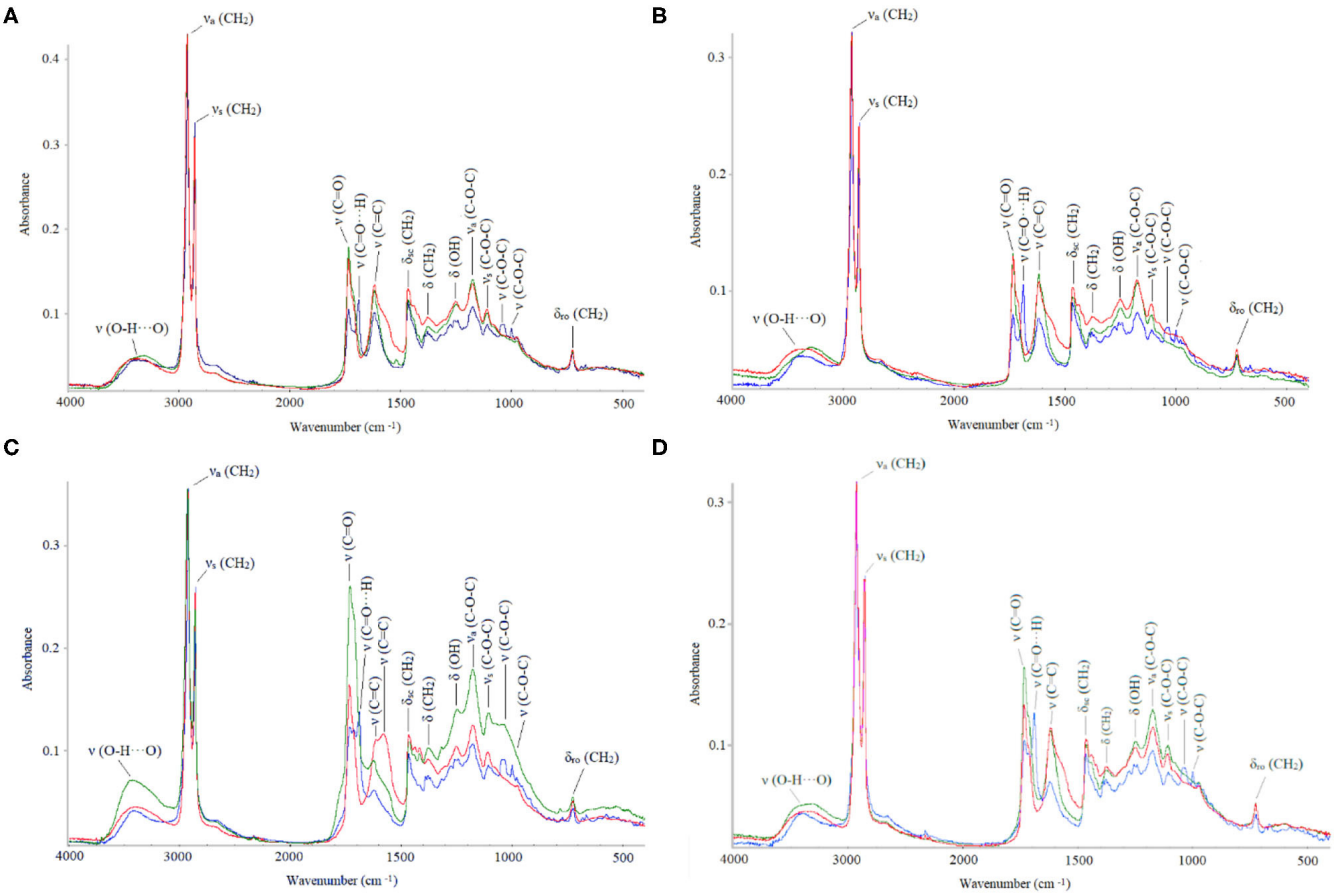
Fourier transform infrared (FTIR) absorbance values (in cm^−1^): natural (blue), dewaxed (green), and combined treatment (red). **(A)** Adaxial young, **(B)** abaxial young, **(C)** adaxial old, and **(D)** abaxial old cuticles.

Absorbance values from the different FTIR bands are shown for the adaxial and abaxial (Figure 3) cuticles, comparing the effect of age and chemical treatments. The results showed that after dewaxing and methanolysis, cuticle composition still included the main groups belonging to cutin, waxes, polysaccharides, and esters. Only one band related to fatty acids (1,688 cm^−1^) was strongly affected in all the groups, and there was a very small effect on glycosidic bonds typical of polysaccharides (1,000 and 1,030 cm^−1^) for the old adaxial side leaves (Figure 3).

The cutin esterification index was significantly lower in the younger leaves (0.3418 ± 0.0899) compared with that in the older leaves (0.4609 ± 0.1791), where the ester bands (1,103, 1,167, and 1,733 cm^−1^) were strongest (Figure 3). The highest index values were recorded for the dewaxed cuticles (0.529 ± 0.1155), and the lowest values were determined for the untreated cuticles (0.263 ± 0.035) and leaf side did not affect index values. Thus, dewaxing was more effective in the young cuticles, and the combined treatment was more effective for the old cuticles.

### Water Loss

**Figure 4:**
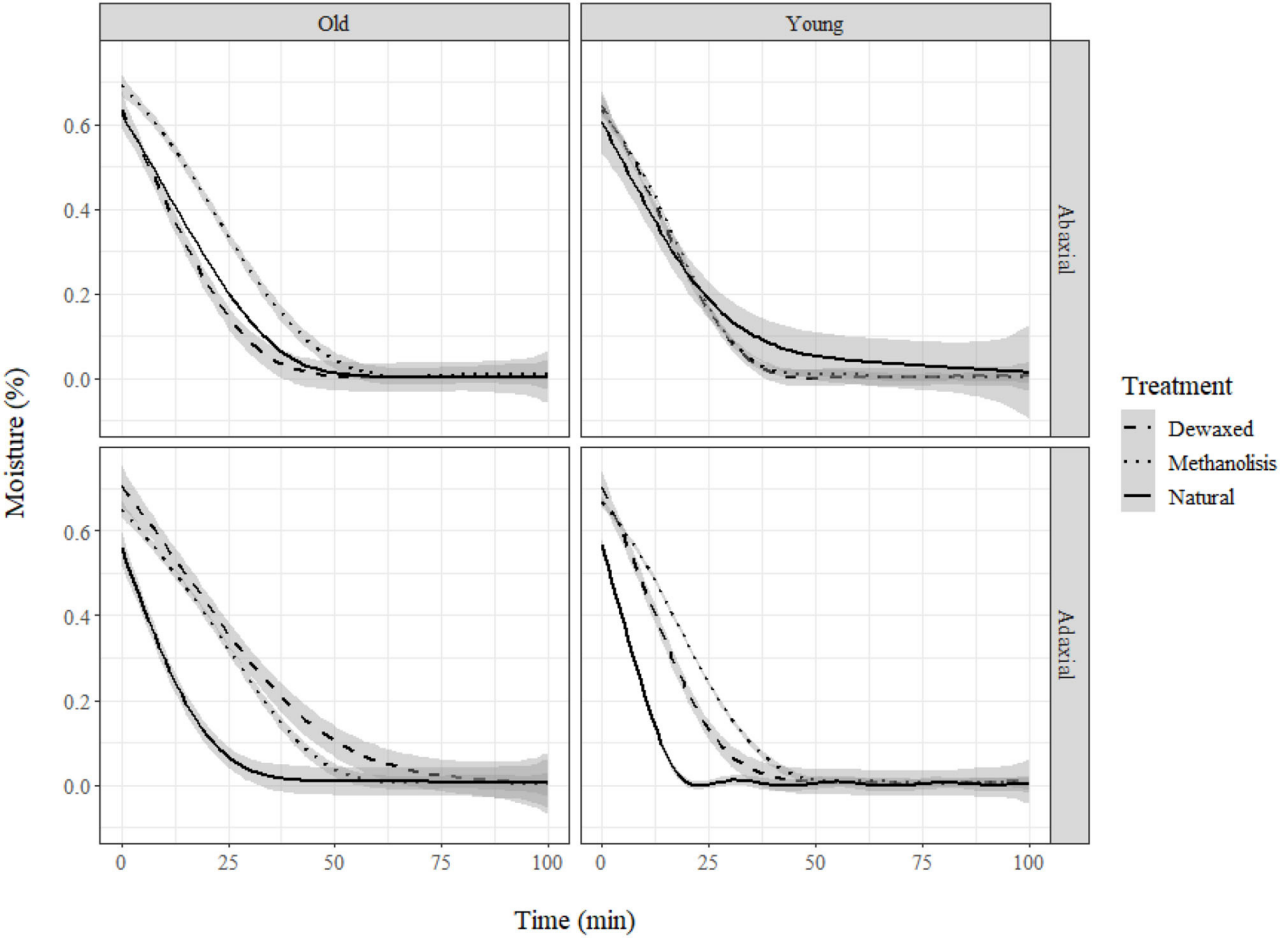
Moisture loss (%) in holly cuticles depending on age, side, and treatment.

Water loss measurements showed that cuticle weight decreased according to an exponential decay model with the equation: *y* = α*e*^β*x*^+θ, where α, β, and θ are coefficients, x is time, and y is weight. Neither temperature nor RH had a significant influence on the trials developed. Age and treatment only led to significant changes for the adaxial leaf cuticles (Figure 4). Treatment increased the total dry time and decreased the water loss rate, while age increased the total dry time. Total absorbed water only varied slightly across the different groups (Table 2).

**Table 2:** Transpiration parameters of holly leaf adaxial cuticles, as affected by chemical treatment and age.

**Age**	**Treatment**	**Beta (β)**	**tseca (min)**	**Absorbed (%)**
Young	Natural	−1.76 ± 0.08a	21 ± 1.4a	124.4 ± 8.4a
	Dewaxed	−2.25 ± 0.11ab	42.5 ± 3.5ab	176.0 ± 20.9a
	D–Methanolysis	−2.65 ± 0.01b	50 ± 0.0b	184.8 ± 16.2a
Old	Natural	−2.06 ± 0.36a	29.5 ± 7.8a	130.2 ± 9.3a
	Dewaxed	−2.91 ± 0.31b	60.0 ± 14.1b	196.0 ± 60.8a
	D–Methanolysis	−2.91 ± 0.07b	57.5 ± 3.5b	170.5 ± 8.8a
Differences	Age	.	^*^	
	Treatment	^**^	^**^	^*^

*Data are means ± SD (n = 30). Within columns and for the same age, values marked with different letters are significantly different according to Tukey's HSD test (P ≤ 0.05)*.

Significance codes: 0 “^***^” 0.001 “^**^” 0.01 “^*^” 0.05 “·” 0.1 “ ” 1.

**Figure 5:**
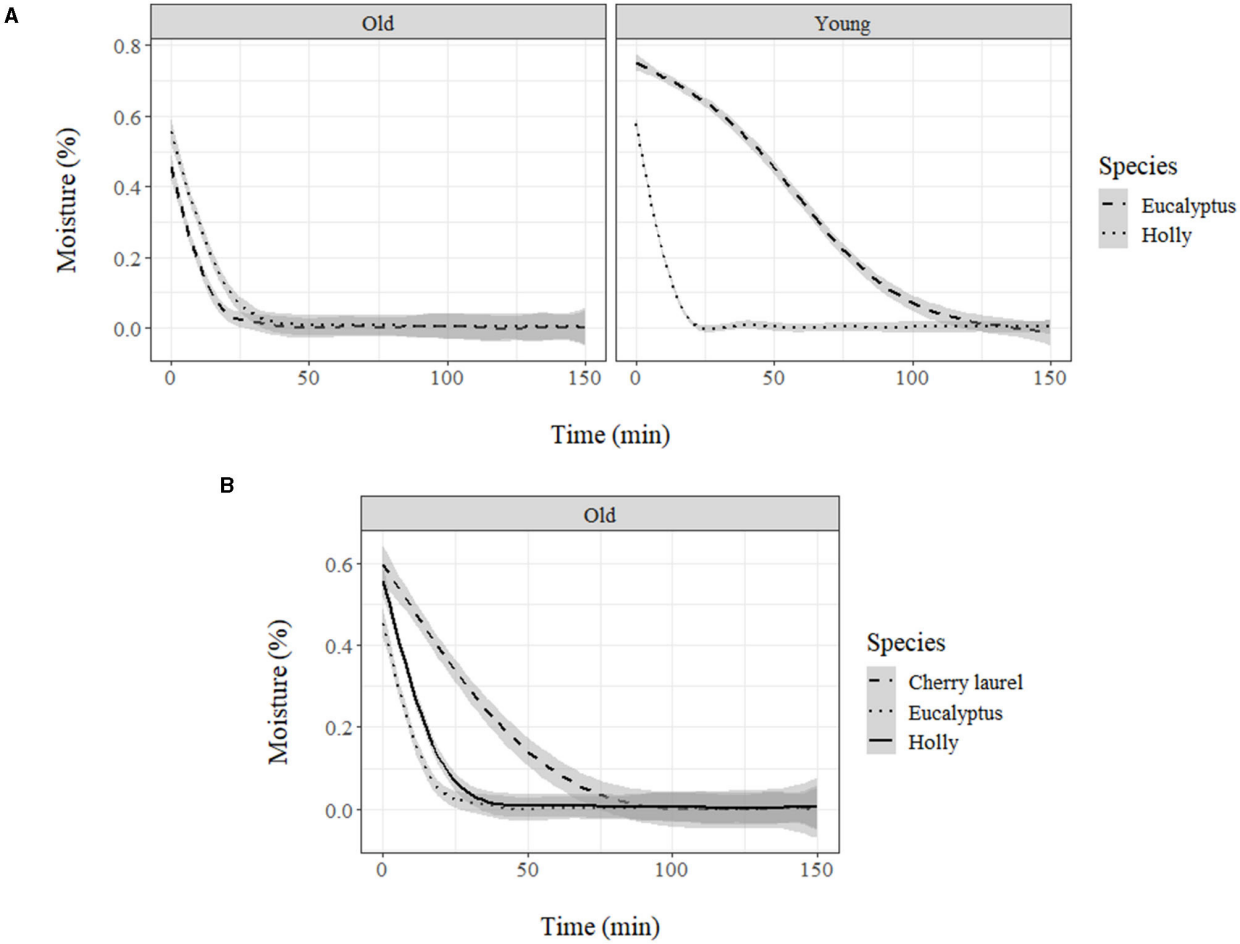
**(A)** Moisture loss (%) of holly and eucalyptus young and old adaxial side cuticles and **(B)** moisture loss (%) of holly, eucalyptus, and cherry laurel old adaxial side cuticles.

When comparing natural adaxial cuticles of the holly and eucalypt, we found that decrease rate varied with age in eucalypt but not in holly (Figure 5). The young Eucalypt cuticles had a steeper decay curve and a higher total dry time, but the total water absorbed was similar in both species (Table 3). We also compared the old natural adaxial cuticles of holly, eucalypt, and cherry laurel. The results showed that the cherry laurel cuticles were significantly different (Figure 5), losing water faster but also taking more time to fully dry than the other two species. Regarding the total water absorbed, holly and cherry laurel absorbed a greater amount of water than eucalypt (Table 4).

**Table 3:** Transpiration parameters of natural adaxial cuticles isolated from holly and blue gum eucalypt leaves.

**Species**	**Age**	**Beta (β)**	**tseca (min)**	**Absorbed (%)**
Holly	Young	−1.75 ± 0.08a	21.0 ± 1.4a	124.4 ± 8.4a
	Old	−2.06 ± 0.36a	29.5 ± 7.8a	130.2 ± 9.3a
Eucalypt	Young	−3.83 ± 0.07a	105.0 ± 7.1a	152.2 ± 22.1a
	Old	−1.90 ± 0.43b	26.5 ± 12.0b	91.1 ± 10.1b

*Data are means ± SD (n = 30). Within columns for the same species, values marked with different letters are significantly different according to Tukey's HSD test (P ≤ 0.05)*.

**Table 4:** Transpiration parameters of natural adaxial cuticles isolated from old holly, blue gum eucalypt, and cherry laurel leaves.

**Species**	**Beta (β)**	**tseca (min)**	**Absorbed (%)**
Holly	−2.06 ± 0.36a	29.5 ± 7.8a	130.2 ± 9.3b
Eucalypt	−1.90 ± 0.43a	26.5 ± 12.0a	91.1 ± 10.1a
Cherry laurel	−3.17 ± 0.45b	75.0 ± 21.2b	152.16 ± 22.1b

*Data are means ± SD (n = 30). Within columns for the same species, values marked with different letters are significantly different according to Tukey's HSD test (P ≤ 0.05)*.

### Foliar Solute Absorption

**Figure 6:**
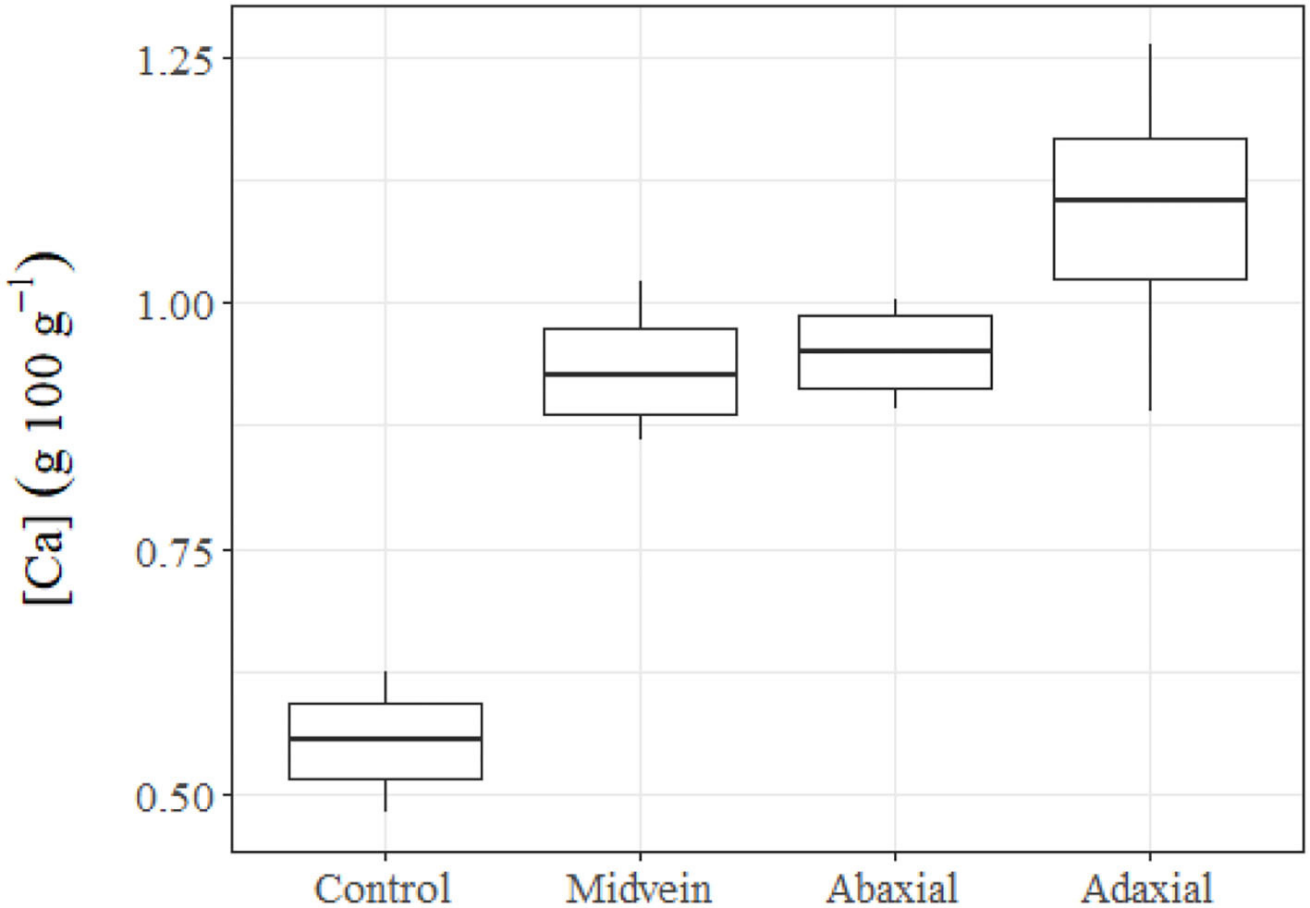
Tissue Ca concentrations of untreated (control) holly leaves compared with leaves treated with 150 mM CaCl_2_ *via* the mid vein, or the adaxial or abaxial leaf side. Holly leaves were sampled 24 h after foliar Ca application.

We found significant differences between the Ca concentration of the untreated leaves of holly and the other three groups supplied with Ca-chloride *via* different leaf parts (Figure 6). The spines were found to have adhesion for water and aqueous solution drops deposited with the syringe of the drop shape analysis system, subsequently, CaCl_2_ drops, which had been carefully hanged to the spines attached leaves with a micro-syringe led to low tissue Ca increases, which were only slightly higher than those found in the untreated control leaves. The highest rate of Ca absorption was recorded after adaxial leaf surface treatment. Calcium solution drop deposition onto the central veins led to tissue Ca concentrations within the same range of leaves treated with Ca *via* the abaxial surface.

## Discussion

In this study, we attempted to establish a link between water loss, cuticular gross chemical composition, and inner structure by comparing the performance of three different species. As noted in previous studies (e.g., Chamel et al., 1991), it is not simple to establish a cause-effect relationship regarding cuticle composition, structure, and water transport, which is likely because of the limited understanding of nanoscale cuticle complexity and variability among the species. These aspects are not easy to trace, because there is an array of methodological and experimental constraints (Fernández et al., 2021). However, using selective chemical treatments and various methodologies can help us gain some ecophysiological insights into the topic (e.g., Chamel et al., 1991; Kerstiens, 2006; Leide et al., 2020). For example, for the adaxial leaf cuticles, we observed no effect of leaf age on the water absorption capacity of holly, while the upper cuticle of the younger eucalypt leaves absorbed more water than the older ones. For both species and leaf age, we observed chemical and structural changes before and after chemical treatment, but the scenario is so complex that we cannot clearly associate cuticular water transport with cuticular chemical composition and ultrastructure. In the following paragraphs, the results obtained will be discussed in light of existing information on cuticle composition and structure in an ecophysiological background, considering also their relevance for foliar water absorption and leaf transpiration.

### Holly Leaf Characterization

Holly leaves have a thick cuticle, which is richer in lipids in older leaves and more abundant in polysaccharides in younger ones. Total cell wall and cuticle thickness were significantly higher in the adaxial side, as previously observed for other species (Gratani et al., 2006; Verdaguer et al., 2012; Vega et al., 2020), and age did not significantly affect thickness, which is in accordance with previous reports (Riederer and Schönherr, 1988; Viougeas et al., 1995; Guzmán-Delgado et al., 2016).

After dewaxing, cuticle thickness decreased, indicating that we had at least altered the composition and structure of some cuticle areas. We extracted 24% of soluble lipids, an amount comparable with other cutan-containing species (Guzmán-Delgado et al., 2016). Age was not a significant factor in terms of soluble lipid extraction, possibly because wax production ceases when the leaf becomes fully expanded, and in the present study, the leaves were sampled well after this developmental stage (Chamel et al., 1992).

Methanolysis was not as effective as expected, because ester bands were identified after this de-esterification treatment, with the occurrence of FTIR bands of similar or higher intensity in natural, dewaxes and de-esterified cuticles. The increase in ester bands could be related to the exposure of internal layers with higher presence of ester bonds. Cutin esterification index was higher in old leaves as part of the cutin polymerization process (Heredia-Guerrero et al., 2020). Cutin esterification index might be related to biomechanical properties of the cuticle, allowing cell enlargement during development according to studies on tomato fruit (España et al., 2014).

### Effect of Cuticle Chemical Treatment

Age and chemical treatment only led to significant changes in the thickness of the adaxial leaf cuticles of holly. Dewaxing affected adaxial side structure (as observed in SEM images) and thickness, resulting in a thinner cuticle but with a better capacity to retain water after a preliminary step of water sorption. A possible explanation for this can be that the extraction of lipids can lead to a more hydrophilic cuticle, hence favoring the retention of water.

Dewaxing and methanolysis affected the adaxial cuticle of the old leaves by reducing fatty acids interacting *via* strong hydrogen bonds (Heredia-Guerrero et al., 2014). Hydroxy fatty acids are highly present in holly cutin (Baker, 1970) but other bands related to cutin and waxes still occurred (720, 1,103, 1,167, 1,244, 1,377, 1,466, 1,688, 1,733, 2,844, and 2,930 cm^−1^) after chemical treatment. The 720-cm^−1^ band is associated with CH_2_ rocking vibration in long chain aliphatic substances (Guo and Bustin, 1998; Heredia-Guerrero et al., 2014) and the 1,377- and 1,466-cm^−1^ bands are bending vibrations of the methyl and methylene groups of fatty acids (Oleszko et al., 2015). Besides, the 1,244- and 1,103-cm^−1^ peaks can be related to C–O modes in secondary alcohols present in cutin (Marechal and Chamel, 1996), while 1,167-cm^−1^ peak can be assignedd to C–O (stretching) vibrations of ester groups, since they contain the majority of C–O groups. The simultaneous presence of aliphatic v(CH_2_) stretching bands at 2,930 and 2,844 cm^−1^ and δ(CH_2_) bands at 1,466, 1,377, and 720 cm^−1^ can be related to esterified aliphatics (Zeier and Schreiber, 1999). Cutin bands were stronger in the adaxial side of both young and old leaves, as also reported by España et al. (2014).

Regarding waxes, Van Genderen et al. (1988) claimed that young leaves of holly were mainly composed of alkanes and esters of long-chain fatty acids. The smaller ester peak observed in older leaves could be a result of fatty acid-esters being used to synthesize alkanes. Furthermore, Niemann and Baas (1985) argued that old leaves have a more polar composition as saturated long-chain hydrocarbons start to accumulate during leaf extension.

Glycosidic bonds ν(C–O–C) were also affected by both treatments but only in the adaxial side of old leaves. Johnson et al. (2007) and Mazurek et al. (2017) associated the 1,030-cm^−1^ peak with cuticular polysaccharides, while Türker-Kaya and Huck (2017) related the peak with O–H and C–OH stretch related to cell wall polysaccharides (arabinan, cellulose). Other bands related to polysaccharides were at 1,000 and 1,622 cm^−1^. Some authors claimed that the peaks at 1,000 cm^−1^are due to aliphatic CH_2_ wagging vibrations in alkenes (Guo and Bustin, 1998), and other reports described the 1,030- and 1,000-cm^−1^ peaks as heavy atoms (CC and CO) of low strength (Adapa et al., 2009) or as aliphatic ethers or alcohols (Chen et al., 2015). Following Stuart (2000), the polysaccharides present in holly are in line with the bands typical for hemicellulose and cellulose. Besides, the peak at 1,622 cm^−1^ may indicate the presence of pectin. We also detected a low-intensity 3,370-cm^−1^ band, which is associated with cutin and polysaccharides, according to Marechal and Chamel (1996), and may indicate the presence of a small number of H-bonds (Heredia-Guerrero et al., 2014).

Overall, the chemical treatments did not lead to major holly cuticle composition changes, as neither dewaxing nor methanolysis significantly affected cuticle ultrastructure. The results point toward the occurrence of cuticular compounds that were highly resistant to chemical treatments, i.e., the so-called cutan. To evaluate this, we compared the results with two species: *Ficus elastica* (rubber tree) and eucalypt, the first with cutan in its cuticle and the second without it. In the case of eucalyptus (Guzmán et al., 2014a), there is a high peak in polysaccharides (3,332 cm^−1^ band), and in non-solubilized long-chain compounds (1,029 cm^−1^) where holly has a much lower band. Besides, there is a decrease in eucalyptus peaks after the treatments. We observed that the spectrum of holly is similar to the spectrum of rubber tree (Guzmán-Delgado et al., 2016), as both species have strong peaks in long chain aliphatics (2,918 and 2,850 cm^−1^) even after being subjected to different treatments. Holly also follows the pattern of cutin polymer described for rubber tree. There are, nonetheless, some differences between them: holly does not have a peak at 468 cm^−1^ and does not decrease esters changing them to the spectral region of the ionized carboxylic groups (Guzmán-Delgado et al., 2016). Furthermore, comparing the absorbance pattern of holly with other two species containing cutan described in the literature (i.e., *A. americana* and *C. miniata*; Heredia-Guerrero et al., 2014), we observed that band distribution adjusted to the pattern of a leaf having cutan. Some studies have described an increase in cutan content as leaves age, improving its mechanical properties (Takahashi et al., 2012; Khanal and Knoche, 2017), and here we aimed to evaluate its potential contribution for dehydration resistance.

### Transport of Water and Solutes

When comparing the rate of cuticular water absorption, we observed that for the holly leaves, age did not significantly affect the percentage of water absorbed or how fast water was subsequently lost. However, in the case of the blue gum eucalypt leaves (a species only having cutin polymer and lacking cutan), age was found to be a main factor, with the young cuticles absorbing more water than the older ones. When comparing between cuticles of older leaves of the three species, we found that both holly and cherry laurel, which contain cutan, absorbed more water than eucalypt. Consequently, the results point toward a potential relationship between cutan and an increased cuticular water sorption capacity. However, more research efforts are required to clarify the effect of cuticle composition and structure on water transport and, ultimately, on plant water economy (Forrester et al., 2010; Fernández et al., 2021).

Evidence was gained that holly leaves can absorb solutes (in this case, modeled with Ca ion) through the adaxial and abaxial surfaces, as well as *via* the mid veins. The amount of Ca absorbed after Ca drop deposit onto the mid veins was within a similar range of leaves treated with Ca through the abaxial and adaxial surfaces. Hence, veins can significantly contribute to the foliar absorption process, as also reported for beech leaves (Bahamonde et al., 2018). Furthermore, we found that the spines also absorbed Ca, but at lower rates than the rest of foliar Ca treatments.

## Conclusions

In this study, we evaluated the effect of chemical treatment on isolated cuticles of young and old holly, cherry laurel, and blue gum eucalypt leaves by analyzing potential ultrastructural and chemical changes. The water sorption and desorption capacity of intact vs. chemically extracted cuticles was also evaluated, and species having cutan (i.e., holly and cherry laurel) were found to sorb more water than eucalypt (only having cutin as lipid polymer). Chemical treatments were not always successful in removing cuticular material, and only minor ultra-structural and chemical changes were recorded for holly leaves. Different solute absorption pathways were tested by the application of a 150-mM CaCl_2_ solution to different leaf areas. Evidence of foliar Ca absorption was gained after Ca-chloride application to both leaf lamina surfaces, the mid veins, and to a lower extent, the spines present in leaf margins. It is concluded that more research is required for the characterization of chemicals and structural features of plant cuticles, and in relation to water and solute transport phenomena.

## Data Availability Statement

The original contributions presented in the study are included in the article/Supplementary Material, further inquiries can be directed to the corresponding author/s.

## Author Contributions

CV collected and analyzed the samples, performed the analysis, wrote the main draft, and edited and revised the article. VF conceived and designed the analysis, analyzed the samples and contributed to the writing, editing, and revision of the article. MV-C and LG contributed to experimental design and revised the draft. All authors accepted the final version of the manuscript.

## Funding

This study was supported by the project S2013/MAE-2760 funded by Comunidad de Madrid. The funding source did not participate in study design, collection, analysis, or interpretation of data, in the writing of the report, or in the decision to submit the article for publication. CV is supported by an FPU predoctoral contract (Ministry of Science and Innovation, Spain).

## Conflict of Interest

The authors declare that the research was conducted in the absence of any commercial or financial relationships that could be construed as a potential conflict of interest.

## Publisher's Note

All claims expressed in this article are solely those of the authors and do not necessarily represent those of their affiliated organizations, or those of the publisher, the editors and the reviewers. Any product that may be evaluated in this article, or claim that may be made by its manufacturer, is not guaranteed or endorsed by the publisher.
