# Supplementary material for: Water Sorption and Desorption of Isolated Cuticles From Three Woody Species With Focus on Ilex aquifolium
Source: Front Plant Sci. 2021 Sep 22;12:728627. doi: 10.3389/fpls.2021.728627 (PMC8522496; doi:10.3389/fpls.2021.728627)

Supplementary Material

# Supplementary Data

Supplementary Material should be uploaded separately on submission. Please include any supplementary data, figures and/or tables. All supplementary files are deposited to FigShare for permanent storage and receive a DOI.

Supplementary material is not typeset so please ensure that all information is clearly presented, the appropriate caption is included in the file and not in the manuscript, and that the style conforms to the rest of the article. To avoid discrepancies between the published article and the supplementary material, please do not add the title, author list, affiliations or correspondence in the supplementary files.

# Supplementary Figures and Tables

For more information on Supplementary Material and for details on the different file types accepted, please see [here](http://home.frontiersin.org/about/author-guidelines#SupplementaryMaterial). Figures, tables, and images will be published under a Creative Commons CC-BY licence and permission must be obtained for use of copyrighted material from other sources (including re-published/adapted/modified/partial figures and images from the internet). It is the responsibility of the authors to acquire the licenses, to follow any citation instructions requested by third-party rights holders, and cover any supplementary charges.

## Supplementary Figures

**
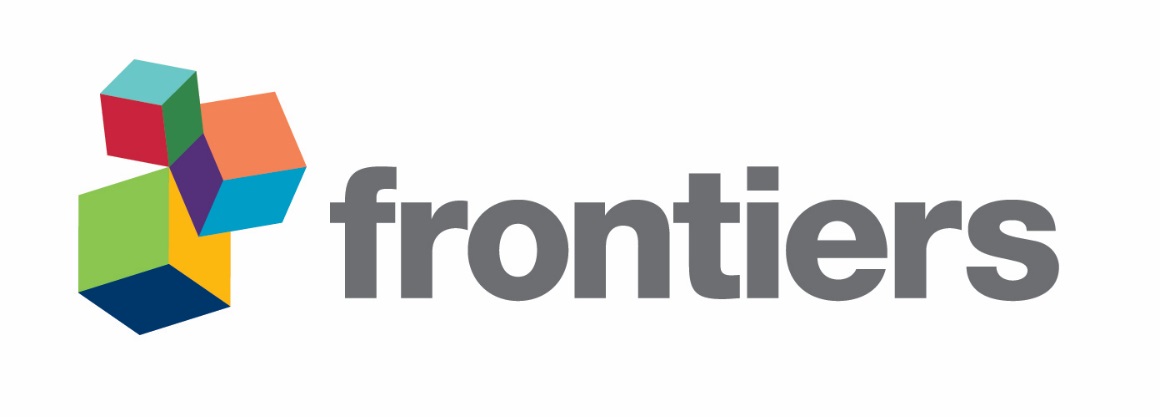
**

**Supplementary Figure 1.** The figure legends are required to have the same font as the main text, 12 point normal Times New Roman, single spaced. Please use a single paragraph for each legend and prepare the figures keeping in mind the PDF layout.

**Supplementary Figure 1.** Cuticle adaxial inner side of (A) natural young, (B) dewaxed young, (C) dewaxed and desterified young, (D) natural old, (E) dewaxed old (F) dewaxed and desterified old.


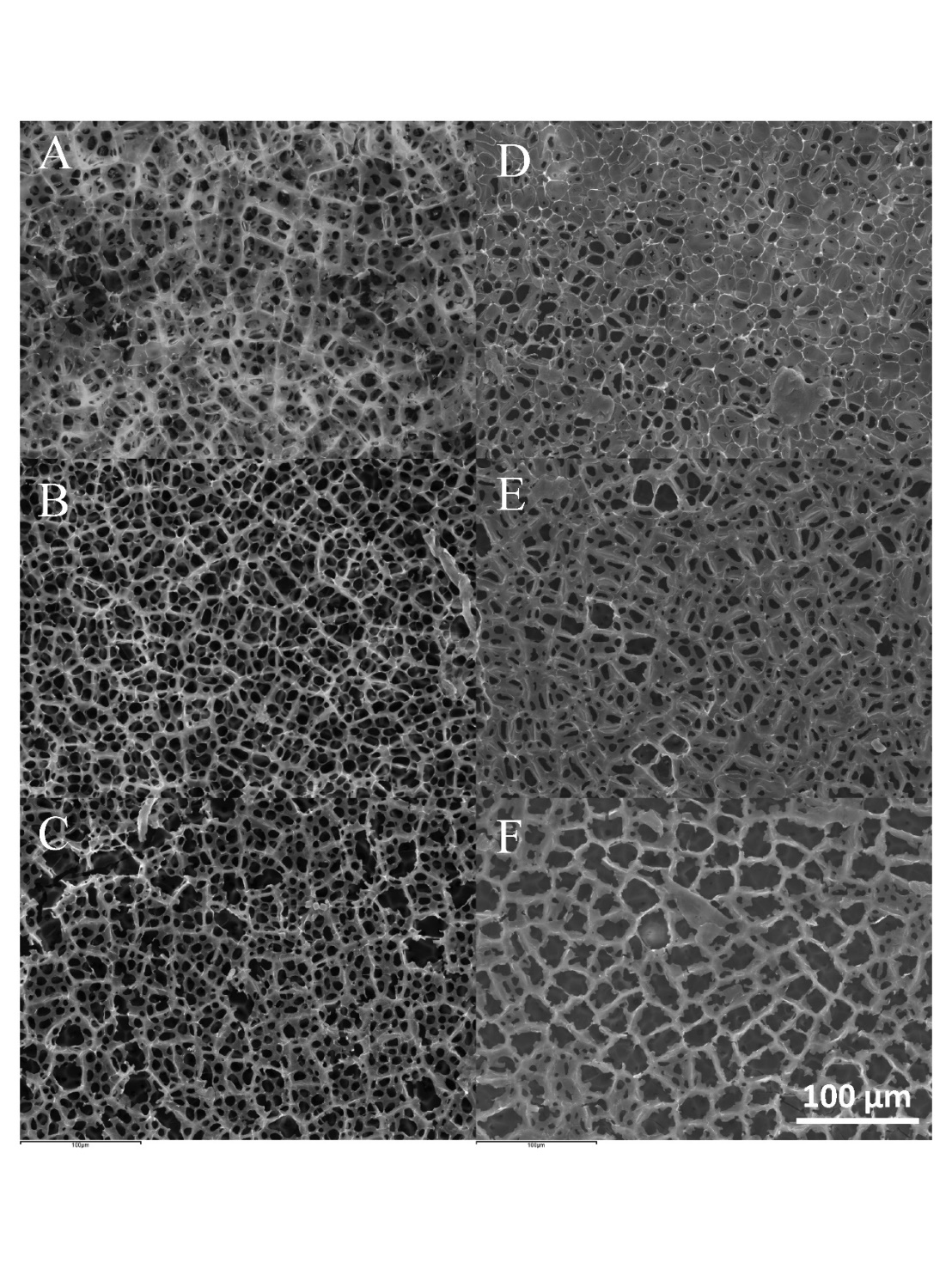


**Supplementary Figure 2.** Cuticle abaxial inner side of (A) natural young, (B) dewaxed young, (C) dewaxed and desterified young, (D) natural old, (E) dewaxed old (F) dewaxed and desterified old.


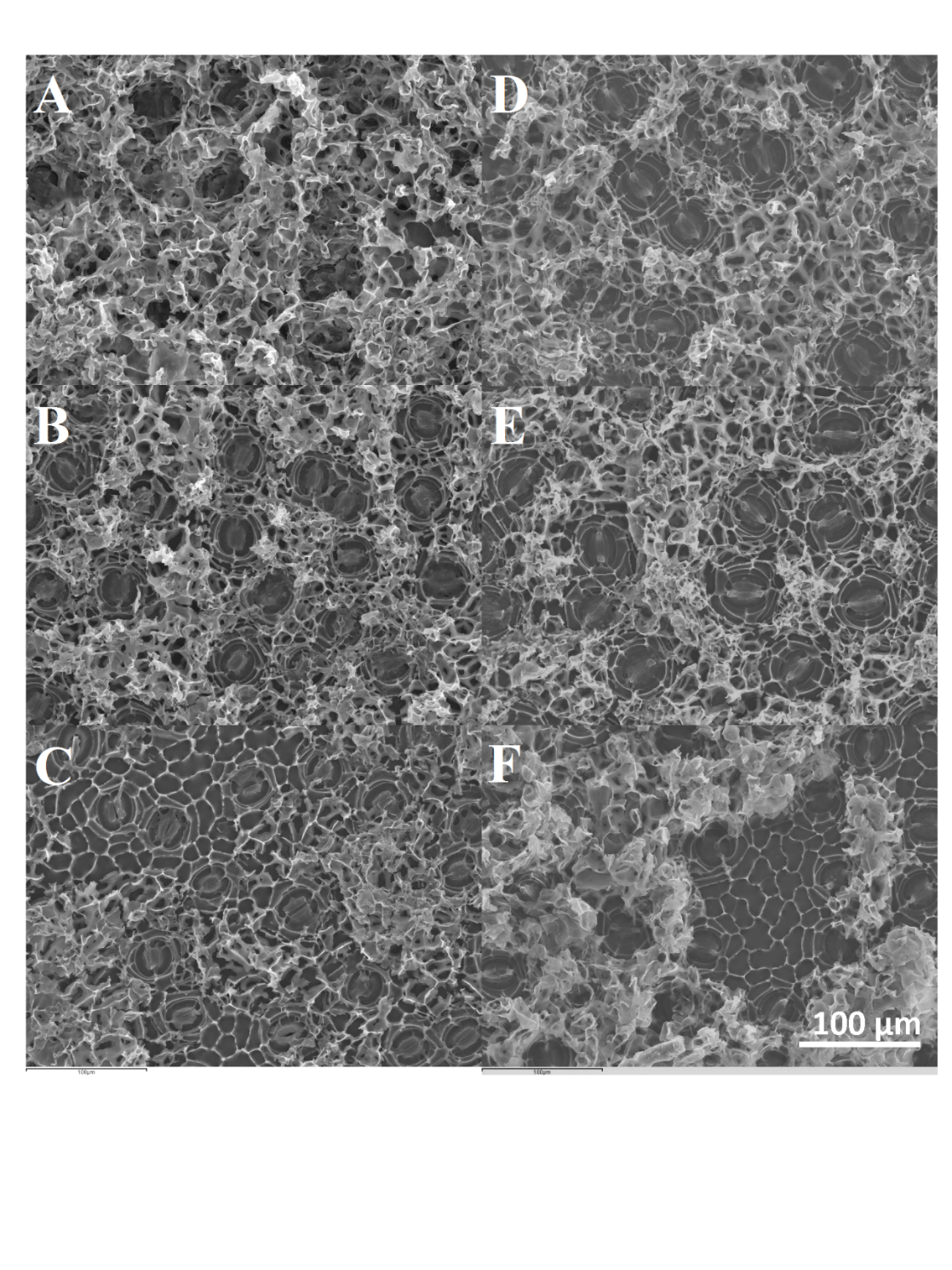

Supplement: Supplementary file 1 [file Data_Sheet_1.docx]
